# Fe_3_O_4_@Au Core–Shell Magnetic Nanoparticles for the Rapid Analysis of *E. coli* O157:H7 in an Electrochemical Immunoassay

**DOI:** 10.3390/bios13050567

**Published:** 2023-05-22

**Authors:** Shayesteh Bazsefidpar, Maria Freitas, Clara R. Pereira, Gemma Gutiérrez, Esther Serrano-Pertierra, Henri P. A. Nouws, María Matos, Cristina Delerue-Matos, María Carmen Blanco-López

**Affiliations:** 1 Department of Physical and Analytical Chemistry & Institute of Biotechnology of Asturias, University of Oviedo, c/Julián Clavería 8, 33006 Oviedo, Spainserranoesther@uniovi.es (E.S.-P.); 2 REQUIMTE/LAQV, Instituto Superior de Engenharia do Porto, Instituto Politécnico do Porto, Rua Dr. António Bernardino de Almeida 431, 4249-015 Porto, Portugalhan@isep.ipp.pt (H.P.A.N.); cmm@isep.ipp.pt (C.D.-M.); 3 REQUIMTE/LAQV, Departamento de Química e Bioquímica, Faculdade de Ciências, Universidade do Porto, 4169-007 Porto, Portugal; clara.pereira@fc.up.pt; 4 Department of Chemical and Environmental Engineering & Institute of Biotechnology of Asturias, University of Oviedo, c/Julián Clavería 8, 33006 Oviedo, Spain; gutierrezgemma@uniovi.es (G.G.); matosmaria@uniovi.es (M.M.)

**Keywords:** pathogenic bacteria, immunomagnetic assay, amperometric biosensor, SPCE, magnetic core–shell nanoparticles

## Abstract

*Escherichia coli* (*E. coli*) O157:H7 is a pathogenic bacterium that causes serious toxic effects in the human gastrointestinal tract. In this paper, a method for its effective analytical control in a milk sample was developed. To perform rapid (1 h) and accurate analysis, monodisperse Fe_3_O_4_@Au magnetic nanoparticles were synthesized and used in an electrochemical sandwich-type magnetic immunoassay. Screen-printed carbon electrodes (SPCE) were used as transducers, and electrochemical detection was performed by chronoamperometry using a secondary horseradish peroxidase-labeled antibody and 3,3′,5,5′-tetramethylbenzidine. This magnetic assay was used to determine the *E. coli* O157:H7 strain in the linear range from 20 to 2 × 10^6^ CFU/mL, with a limit of detection of 20 CFU/mL. The selectivity of the assay was tested using *Listeria monocytogenes* p60 protein, and the applicability of the assay was assessed by analyzing a commercial milk sample, demonstrating the usefulness of the synthesized nanoparticles in the developed magnetic immunoassay.

## 1. Introduction

*E. coli* O157:H7 is a pathogenic bacteria strain that causes severe illness in humans through contaminated food or water. This bacterium is a Shiga-like toxin producer and causes severe foodborne diseases, especially in young children, the elderly, and patients with sensitive immune systems [1,2]. The World Health Organization (WHO) states that intoxication by *E. coli* O157:H7 could lead to serious conditions such as hemorrhagic diarrhea and acute kidney failure. The transmission of this strain mainly occurs through the consumption of undercooked or contaminated foods and liquids. For this reason, several countries have implemented guidelines concerning the identification of *E. coli* O157:H7 in food products to ensure public safety [3,4]. Therefore, monitoring *E. coli* O157:H7 is a challenge in the food safety field.

The rapid detection of *E. coli* O157:H7 at low concentrations is also a big concern in other fields, such as in environmental applications, medicine, and pharmacy [5,6]. Various conventional microbiological strategies are commonly applied for the analysis and detection of pathogenic bacteria, including Tissue Culture Plate (TCP), Plate Count Enumeration Method, Quartz Crystal Micro-balance resonators (QCM), and Polymerase Chain Reaction (PCR). Nevertheless, these traditional methods are significantly time-consuming, labor-intensive, and require specialized staff [7,8]. 

Electrochemical biosensors and bioassays are analytical tools with several intrinsic attractive features, namely selectivity, sensitivity, speed in response, low production cost, simplicity of construction, facile operation, and ease of miniaturization; moreover, they can potentially reach low limits of detection (LOD) [9,10]. Electrochemical immunosensors have been used for their high selectivity, accuracy, precision, and capability to identify bacteria [11]. Among the different immunosensing configurations, sandwich assays have been widely employed [12]. This assay type allows the detection of an antigen through its interaction with antibodies on a transducer, such as a screen-printed carbon electrode (SPCE), to convert the biochemical reaction into a measurable electrical signal [13,14]. SPCEs have been proven to be convenient platforms for on-site and rapid analysis [14]. Chronoamperometry is a sensitive electrochemical detection technique that involves the application of a fixed potential to an electrochemical cell and the recording of the current intensity during a pre-established period [15,16]. Compared to other detection methods, chronoamperometry is preferred because of its simplicity and superior signal-to-noise ratio, allowing the detection of the analyte at low concentrations [15].

In recent years, several nanomaterials have been used in the construction of electrochemical biosensors and bioassays because of their electrocatalytic activity and high surface area, improving the sensitivity of the analysis, and in many cases, the selectivity, reducing electrochemical interferences [17]. Among the different classes of nanomaterials, superparamagnetic iron oxide nanoparticles (SPIONs) have received significant attention for the immobilization of many biomolecules due to their easy manipulation with an external magnetic field, biocompatibility, and high surface area-to-volume ratio [18]. SPIONs allow the sample concentration and elimination of interferents with efficient washing steps due to their magnetic properties, thus improving the biosensors’ performance [14,19]. These nanoparticles, with a typical diameter lower than 20 nm, rapidly respond to an external magnetic field and can be easily redispersed after the magnet is removed due to their superparamagnetic properties at room temperature [19,20]. This property can also increase the probability of detecting the target analyte in a small volume of sample through electrochemical biosensors [11]. However, these uncoated nanoparticles are prone to oxidation and tend to aggregate over time in a liquid medium. To overcome these drawbacks, iron oxide nanoparticles are often coated with organic or inorganic layers [21]. By coating SPIONs with inorganic layers such as gold, functional thiol groups can easily be introduced on their surface for covalent reaction with active biomolecules, such as antibodies, aptamers, or nucleic acids. The gold coating also increases the stability of the nanoparticles [22].

In this work, an electrochemical magnetic nanoparticle-based sandwich-type immunoassay for the analysis of *E. coli* O157:H7 was developed. An SPCE was used as the transducer, and core–shell Fe_3_O_4_@Au magnetic nanoparticles (MNPs) were synthesized and used for the first time for this purpose. A sandwich immunocomplex was formed on the MNPs that were attracted to the surface of the SPCE by a small magnet. After the addition of the enzymatic substrate, chronoamperometry was used to record the analytical signal at a very low potential (0 V), reducing possible interferences from the sample matrix. The immunoassay’s selectivity and applicability were evaluated by analyzing *Listeria monocytogenes* p60 protein and a food (milk) product, respectively. The results demonstrated the adequate performance and usefulness of the assay for the fast determination (1 h) of *E. coli* O157:H7 in a 40-µL sample, which are significant advantages when compared with the traditional methods.

## 2. Materials and Methods

### 2.1. Equipment, Reagents, and Solutions

Mouse monoclonal antibodies to *E. coli* O157:H7 were purchased from MyBioSource (San Diego, CA, USA). A secondary polyclonal antibody conjugated with HRP was purchased from Dako (Carpinteria, CA, USA). Recombinant *Listeria monocytogenes* p60 protein (LM p60 protein), expressed in *E. coli*, was obtained from Adipogen Life Sciences (Füllinsdorf, Switzerland). 

The *E. coli* O157:H7 capture antibody, the *E. coli* O157:H7 detection antibody, and the secondary HRP-labeled antibody are abbreviated as Ab-C, Ab-D, and HRP-Ab, respectively.

*N*-Hydroxysuccinimide (NHS), 1-ethyl-3-[3-dimethylaminopropyl]carbodiimide hydrochloride (EDC), 2-(*N*-morpholino)ethanesulfonic acid (MES), bovine serum albumin (BSA), phosphate-buffered saline (PBS), tris(hydroxymethyl)aminomethane (Tris), 3,3′,5,5′-tetramethylbenzidine liquid substrate (TMB-H_2_O_2_ K-Blue reagent), ethanolamine (EA), potassium dihydrogen phosphate, potassium hydrogen phosphate trihydrate, sodium chloride, iron(III) acetylacetonate ([Fe(acac)_3_], 99%), tetrachloroauric(III) acid trihydrate (HAuCl_4_·3H_2_O, ≥99.9%, Au 48.5–50.25%), absolute ethanol (analytical grade), anhydrous toluene (99.8%), thioctic acid (TOA), 1-methyl-2-pyrrolidinone (NMP, ≥99.0%), Tween^®^ 20, oleylamine (80%), 1-hexadecanol (96%), oleic acid (90%), and 6-mercapto-1-hexanol (MCH) were obtained from Sigma-Aldrich (St. Louis, MO, USA).

The following solutions were used in this work: 10 mM MES buffer, pH 6 (B1), for activation of the carboxylic groups; 10 mM PBS containing 137 mM NaCl, pH 8.3 (B2), for preparing the blocking solution; and 10 mM PBS containing 137 mM NaCl, pH 7.4 (B3), to prepare working solutions and the antigen (*E. coli* O157:H7). For the washing steps, Tween^®^ 20 (0.01%), a surfactant used to prevent non-specific biomolecule binding and to reduce background signals, was included in the washing buffers (B1-T, B2-T, B3-T). All the reagents were prepared using Milli-Q ultrapure water (resistivity 18.2 MΩ·cm, at 25 °C) unless stated otherwise.

All the steps of the sandwich immunomagnetic assay were performed at room temperature, under continuous mixing (950 rpm) using a HulaMixer (Thermo Fisher Scientific, Oslo, Norway), and protected from light. The washing steps consisted of the addition of 100 μL of the desired buffer containing Tween-20, followed by additional stirring for 2 min. A DynaMagTM-2 magnetic rack (Life Technologies, Oslo, Norway) was used for the MNPs’ attraction and separation of the supernatant in the various steps of the assay.

The chronoamperometric measurements were performed on SPCEs (DRP-110, Metrohm DropSens, Oviedo, Spain) that were connected (connector cable, DRP-CAC, Metrohm DropSens, Oviedo, Spain) to a potentiostat/galvanostat (PGSTAT 101, Metrohm Autolab, Utrecht, The Netherlands). A specific base containing a 4 mm diameter magnet was used to support the SPCE for precise magnetic attraction of the MNPs to the working electrode (WE) of the SPCE. All the measurements were performed in triplicate.

### 2.2. Preparation of E. coli O157:H7

*E. coli* O157:H7 was cultured in 50 mL of Tryptic Soy Broth (TSB) medium at 37 °C and an agitation speed of 240 rpm overnight. Next, the culture medium, including bacteria, was centrifuged at 1000 rpm for 5 min, and then the pellet was resuspended in 5 mL of PBS. To inactivate the bacteria for safe handling, the cells were heated at 100 °C for 15 min. Samples were frozen until use.

### 2.3. Fe_3_O_4_@Au Synthesis and Functionalization

The Fe_3_O_4_@Au MNPs were synthesized and functionalized following our previously reported method [19]. It consisted of (i) the synthesis of Fe_3_O_4_ MNPs by thermal decomposition, followed by (ii) the coating of the Fe_3_O_4_ MNPs with a gold shell by chemical reduction of Au(III) using oleylamine as reducing and capping agent, and (iii) the functionalization of the resulting Fe_3_O_4_@Au core–shell nanoparticles with a mixture of MCH and TOA (3:1 V/V). Briefly, NMP (35 mL), oleic acid (0.3 M), 1-hexadecanol (0.3 M), and oleylamine (0.3 M) were mixed and heated to 200 °C (under stirring and inert atmosphere). Then, [Fe(acac)_3_] (0.15 M, 10 mL) was quickly added to this solution, and stirring was continued for 1 h. Then, the reaction mixture was cooled and kept under stirring overnight. The resulting MNPs were precipitated by adding ethanol (50 mL), and the material was washed several times with ethanol (to remove the excess of oleylamine and oleic acid) and redispersed in anhydrous toluene (5 mL).

The Fe_3_O_4_ MNPs were coated with a gold shell using a Fe_3_O_4_:HAuCl_4_·3H_2_O molar ratio of 1:7. Briefly, the Fe_3_O_4_ MNP dispersion (1.25 mL) was diluted with anhydrous toluene (20 mL) and heated to 100 °C under an inert atmosphere. Subsequently, a solution containing HAuCl_4_.3H_2_O, oleylamine (7.07 mL), and anhydrous toluene (35 mL) was added dropwise under vigorous stirring to the pre-heated dispersion. The reaction mixture was stirred for 1 h. Then, the system was cooled to room temperature, and absolute ethanol (50 mL) was added. The coated nanoparticles were magnetically separated and washed several times with ethanol. The final nanomaterial was redispersed in anhydrous toluene (10 mL), and a dark red-purple dispersion was obtained.

The Fe_3_O_4_@Au concentration was determined and expressed in mg/mL. The functionalization of the Fe_3_O_4_@Au MNPs (4 mg/mL) was performed using ethanolic solutions of MCH (0.1 M) and TOA (0.1 M). The ethanolic thiol mixture with a volume ratio of 3:1 was added to the Fe_3_O_4_@Au dispersion and kept under stirring overnight. Then, the supernatant was removed (using a magnet), and the MNPs were dispersed in B1.

### 2.4. Magnetic Nanoparticles Characterization

The MNPs were characterized by transmission electron microscopy (TEM) on a JEOL-2000 Ex II TEM (Japan) equipment (JEOL, Tokyo, Japan). The procedure involved dispersing the samples in toluene under sonication and subsequently immersing a carbon-coated 200-mesh copper grid in the suspension, followed by air-drying. The average particle sizes and size distributions were calculated from the diameters of at least 100 particles randomly selected from the TEM micrographs. The dynamic light scattering (DLS) measurements were performed at 25 °C on a Zetasizer Nano ZS (Zetasizer, Malvern, UK).

### 2.5. Immobilization of Capture Antibody on Fe_3_O_4_@Au

The immobilization of the Ab-C on the Fe_3_O_4_@Au MNPs consisted of the following steps: 50 mg of Fe_3_O_4_@Au MNPs (0.25 mg/mL) functionalized with MCH-TOA were washed, followed by the separation of the modified Fe_3_O_4_@Au MNPs using the magnetic rack and disposal of the supernatant after 1 min. Then, the free carboxylic groups of the modified Fe_3_O_4_@Au MNPs were activated by mixing with 100 μL solution of EDC (200 mM) and NHS (50 mM) prepared in 500 μL of B1 buffer for 15 min under stirring. The supernatant was removed, and 100 μL of Ab-C (10 μg/mL, in B1) were added for 1 h, followed by a washing step with B1-T and B2-T. Finally, 100 μL of EA (0.1 M, in B2) were added and incubated for 10 min to block the unreacted carboxylic groups. Subsequent washing steps with B2-T and B3-T were carried out, and the Ab-C-modified Fe_3_O_4_@Au MNPs were finally dispersed in B3-T and stored at 4 °C until use.

### 2.6. Immunoassay Procedure

**Figure 1:**
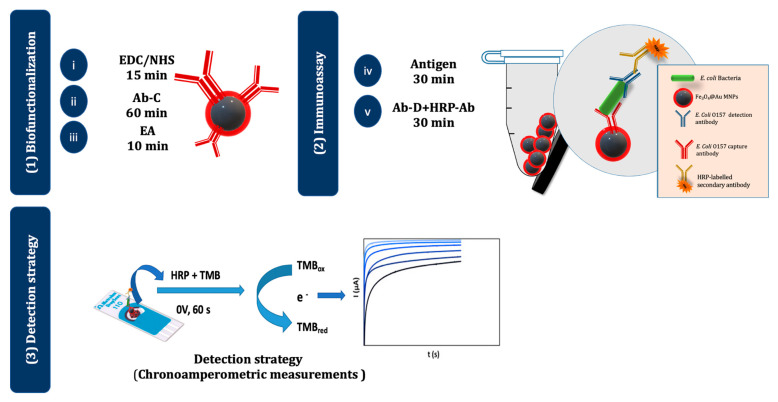
Schematic representation of the electrochemical magnetic immunoassay. (**1**) The surface of the Fe_3_O_4_@Au MNPs was (i) activated with EDC/NHS, (ii) modified with Ab-C, and (iii) blocked with EA. (**2**) The assay was performed by adding (iv) the antigen (*E. coli* O157:H7) and (v) an Ab-D+ HRP-Ab mixture. (**3**) The MNPs were then magnetically attracted to the WE of the SPCE, TMB was added, and chronoamperograms were recorded.

Figure 1 illustrates the different steps of the biofunctionalization, optimized immunoassay, and electrochemical detection strategy. In the optimized assay, a 10-μL aliquot of the Ab-C-modified Fe_3_O_4_@Au MNPs dispersion was taken, the supernatant was removed, and 100 μL of an *E. coli* O157:H7 standard/sample solution was added and incubated for 30 min. Then, 100 μL of a solution containing both Ab-D (5.0 μg/mL) and HRP-Ab (initial concentration: 1 g/L; 2000× dilution in B3) were added and incubated for 30 min. A final washing step was performed with B3-T, and the suspension was maintained in B3-T. This suspension (10 μL) was placed on the SPCE, the MNPs were attracted to the working electrode (WE) with a magnet, and the supernatant was removed. The biorecognition event was monitored by adding the enzymatic substrate TMB (40 μL, 1 min, and absence of light) and subsequently recording a chronoamperogram (at 0 V for 1 min). During the enzymatic reaction, HRP catalyzes the oxidation of TMB, which is then detected by chronoamperometry [23].

### 2.7. Milk Sample Analysis

To evaluate the applicability of the proposed assay, a milk sample was purchased from a local supermarket (Central Lechera Asturiana, Asturias, Spain) and used without any pretreatment. The milk sample was spiked with different *E. coli* O157:H7 concentrations (2 × 10^3^, 2 × 10^2^, and 2 × 10^1^ CFU/mL), diluted (1:10), and analyzed. Control samples (non-spiked milk) were also included in the analysis.

## 3. Results and Discussion

### 3.1. Fe_3_O_4_@Au MNP Synthesis and Characterization

**Figure 2:**
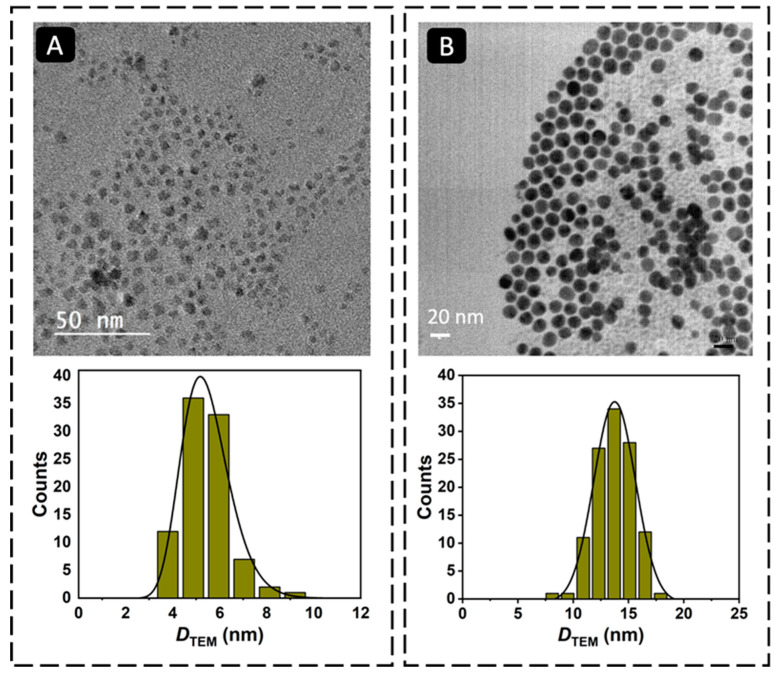
TEM micrographs and particle size distribution histograms of (**A**) Fe_3_O_4_ MNPs (magnification: 10,000×) and (**B**) Fe_3_O_4_@Au MNPs (magnification: 13,000×).

**Figure 3:**
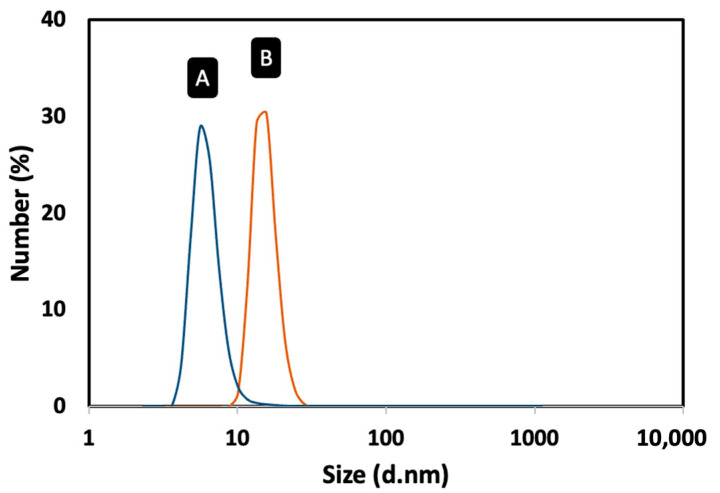
Solvodynamic particle size distribution profiles of the MNPs dispersed in toluene obtained by DLS: (**A**) Fe_3_O_4_ MNPs; (**B**) Fe_3_O_4_@Au MNPs.

The magnetic Fe_3_O_4_ cores were synthesized by the thermal decomposition method [19]. To prevent the oxidation of the Fe_3_O_4_ MNPs and to use them as platform for the development of the magnetic immunoassay, they were coated with a gold shell. The coating process involved a gradual deposition of gold onto the surface of the Fe_3_O_4_ MNPs by a controlled temperature-induced reduction of Au(III) to Au(0) using oleylamine. In this process, oleylamine functions both as a reducing agent and as a stabilizer of the resulting core–shell MNPs, preventing the agglomeration of the nanoparticles and maintaining their stability [24]. Figure 2 shows the TEM micrographs of Fe_3_O_4_ and Fe_3_O_4_@Au MNPs. The TEM images revealed that the Fe_3_O_4_ MNPs were well dispersed, presenting a log-normal particle size distribution and an average particle size of 5.4 nm (*σ* = 1.0). The Fe_3_O_4_@Au MNPs had a nearly spherical shape and a particle size of 13.7 ± 1.9 nm, confirming the coating of the Fe_3_O_4_ cores with gold (Table 1). Dynamic light scattering (DLS) measurements were carried out to confirm the coating of the Fe_3_O_4_ MNPs with gold and obtain information on the colloidal stability of the nanomaterials (Figure 3). The Fe_3_O_4_ MNPs dispersed in toluene presented an average solvodynamic diameter of 5.6 nm, while the Fe_3_O_4_@Au MNPs showed an average solvodynamic diameter of 15.7 nm (Table 1). In both cases, the solvodynamic particle sizes are consistent with the corresponding particle sizes obtained by TEM, indicating the lack of agglomeration of the particles in the solution.

### 3.2. Optimization of Experimental Conditions

For the development of a successful immunoassay, the optimization of the experimental parameters is fundamental [25,26]. Therefore, the variables of interest, such as the working solution, incubation time, and reagents’ concentrations, were optimized according to their relevance to the immunoassay. The selected values were chosen according to the signal-to-blank ratio (S/B) using the current intensity values measured in the absence and in the presence of 2 × 10^6^ CFU/mL of *E. coli* O157:H7. The obtained data correspond to the average and standard deviation of three replicates.

**Figure 4:**
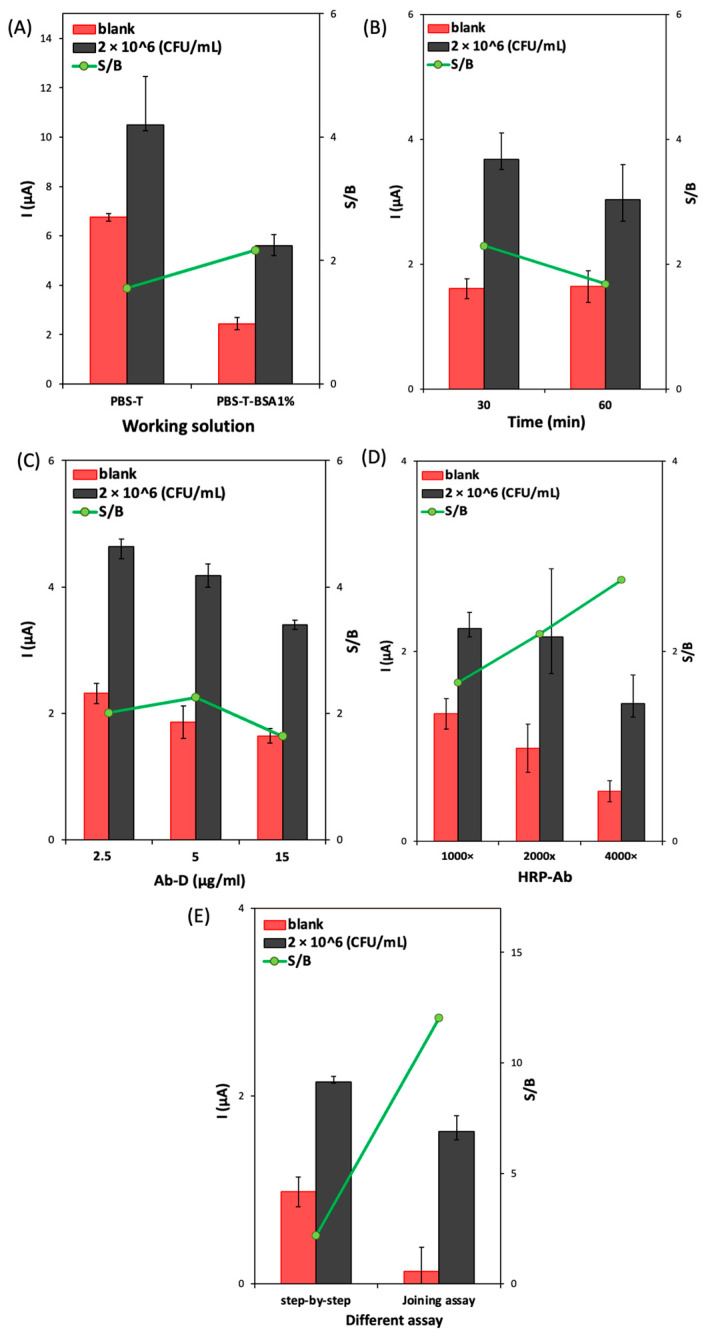
Optimization of (**A**) the working solution (B3-T and B3-T with BSA 1%); (**B**) the incubation time for the step-by-step assay; (**C**) the Ab-D concentration (2.5, 5.0, and 15 μg/mL); (**D**) the HRP-Ab dilution (1000×, 2000×, and 4000×); and (**E**) comparison of step-by-step assay and joined step assay. Experimental conditions: Ab-C-modified Fe_3_O_4_@Au MNPs volume: 10 μL; Ab-C concentration: 10 μg/mL; and *E. coli* O157:H7 concentration: 2 × 10^6^ CFU/mL.

To obtain selective detection of the target bacteria, it is necessary to minimize the background signal related to the non-specific reaction of biomolecules on the sensor platform. Therefore, the effect of adding BSA to the working solutions was evaluated. B3-T and B3-T-BSA 1% (m/V) were tested and compared using the following conditions: Ab-C-modified Fe_3_O_4_@Au MNPs volume: 10 μL; Ab-C concentration: 10 μg/mL; *E. coli* O157:H7 concentration: 2 × 10^6^ CFU/mL; Ab-D concentration: 5 μg/mL, HRP-Ab: 2000× dilution; and incubation time: 60 min. As can be observed in Figure 4A, the S/B ratio improved when the immune reactions in different steps were performed in the working solution containing BSA 1% (m/V). The addition of BSA clearly reduced the blank signal more than the signal obtained in the presence of bacteria, improving the S/B ratio. Therefore, B3-T-BSA 1% (m/V) was selected to proceed with the studies.

To improve the total assay time, the ‘step-by-step assay’ approach was tested using different incubation times (30 or 60 min): incubation of (1) bacteria, 30 min; Ab-D, 30 min; and HRP-Ab 30 min; (2) bacteria, 60 min; Ab-D, 60 min, and HRP-Ab, 30 min. In Figure 4B, it is possible to observe that the increase in the incubation time (assay 2) decreased the S/B ratio. Therefore, an incubation time of 30 min for each biomolecule (bacteria, Ab-D, and HRP-Ab) was used in the subsequent studies.

The Ab-D concentration (2.5, 5.0, and 15 μg/mL) was optimized under the previously mentioned conditions. Figure 4C shows that when the Ab-D concentration increased from 2.5 to 5.0 μg/mL, the S/B ratio also increased; however, for the highest concentration (15 μg/mL), the S/B ratio decreased, which indicates that saturation was attained. Therefore, 5.0 μg/mL of Ab-D was chosen to proceed with the development of the assay. The dilution of the HRP-Ab (1000×, 2000×, and 4000×) was optimized under the previously optimized conditions. The obtained results (Figure 4D) show that higher dilutions led to an increase in the S/B ratio but with lower bacteria and blank signals. Therefore, the intermediate dilution of 2000× was considered adequate.

Regarding the optimization of the total assay time, a combined (joined) step protocol, comprising the preincubation of Ab-D (5.0 μg/mL) and HRP-Ab (2000×) in a single solution (15 min before use in the assay), was compared with the step-by-step procedure. The obtained data (Figure 4E) shows that the joined step strategy led to the highest S/B ratio. It also decreased the assay time from 1.5 h to 1 h and the number of incubation steps, so this procedure was adopted for the following studies.

### 3.3. Analytical Performance of the Immunoassay for E. coli O157:H7 Analysis

**Figure 5:**
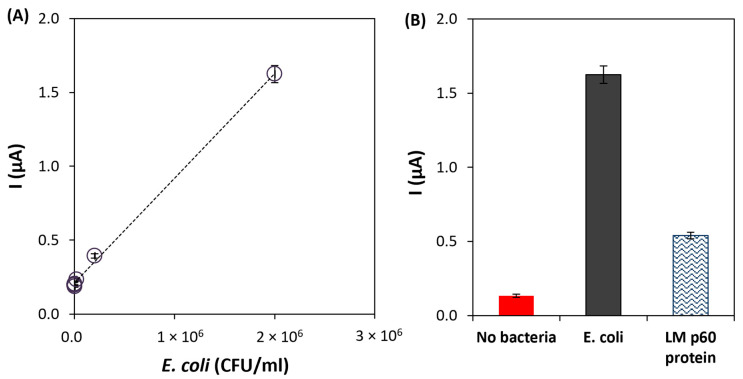
(**A**) Calibration line of the developed immunoassay for the analysis of *E. coli* O157:H7. (**B**) Results of the selectivity studies using LM p60 protein (1000 ng/mL). Experimental conditions: Ab-C-modified Fe_3_O_4_@Au MNPs volume: 10 μL; Ab-C concentration: 10 μg/mL; Ab-D concentration: 5 μg/mL; and HRP-Ab dilution: 2000× dilution.

To test the performance of the developed immunoassay, different solutions of *E. coli* O157:H7 were prepared (2 × 10^1^, 2 × 10^2^, 2 × 10^3^, 2 × 10^4^, 2 × 10^5^, 2 × 10^6^, and 2 × 10^7^ CFU/mL) and analyzed using the optimized conditions. The calibration straight was established between 2 × 10^1^ and 2 × 10^6^ CFU/mL, with the following linear regression equation: *i* (µA) = 7.1 × 10^−7^ [*E. coli*] (CFU/mL) + 0.21, r^2^ = 0.998, *n* = 6 (Figure 5A). The limit of detection (LOD = 20 CFU/mL) was calculated according to the equation 3.3 × *σ*/*S*, where *σ* is the standard deviation of the intercept, and *S* is the slope of the calibration line. Furthermore, the coefficient of variation of the method (9.9%) demonstrated the good precision of the method. The analytical characteristics allow us to conclude that the developed immunoassay could be applied to the analysis of *E. coli* in food samples.

Furthermore, the selectivity of the immunoassay was tested against a cell surface protein (p60) of *Listeria monocytogenes* (1000 ng/mL) that is secreted in large quantities into growth media. As shown in Figure 5B, the current intensity obtained for *E. Coli* O157:H7 was significantly higher than the one obtained for the *Listeria monocytogenes* protein, thus demonstrating that good selectivity was achieved. According to the obtained results, an additional washing step or the adjustment of the Tween-20 amount in the washing buffer could allow a more effective removal of the non-target protein, reducing the corresponding signal. However, this would lead to a more laborious procedure.

### 3.4. Milk Sample Analysis

To assess the accuracy of the assay’s results, liquid milk was selected to evaluate the application of the proposed assay for rapid detection (1 h assay) of *E. coli* O157:H7 in foods. The sample was prepared as explained in Section 2.7, “milk sample analysis”, and spiked with different concentrations of *E. coli* O157:H7 (0; 2 × 10^1^, 2 × 10^2^, and 2 × 10^3^ CFU/mL). *E. coli* O157:H7 was only quantifiable in the 2 × 10^2^ and 2 × 10^3^ CFU/mL solutions. Acceptable recoveries for these concentrations were obtained (110% and 125%) with adequate precision (the coefficient of variation of the results (CV) was lower than 3%) (Table 2). Although a LOD of 20 CFU/mL was achieved in buffer solution, bacteria analysis in food samples (such as milk) is always a challenge because of the complexity of the sample matrix. Therefore, for lower bacteria concentrations, a lower analytical signal than the one obtained for the LOD in buffer solution was obtained.

### 3.5. Comparison with Other Electrochemical Immunoassays for the Determination of E. coli

Table 3 shows a comparison between the magnetic immunoassay developed in this work and reported electrochemical sensors/assays for *E. coli* analysis. The Fe_3_O_4_@Au MNPs have several advantages due to their unique combination of SPIONs and a gold shell. Firstly, the magnetic cores allow the efficient collection of the analyte during the assay, which enhances the sensitivity of the assay and reduces the signal background signal because of the elimination of possible interferents during the washing step. Secondly, the gold shell of the Fe_3_O_4_@Au MNPs provides several advantages, such as increased biocompatibility, chemical stability, and ease of functionalization. The gold shell makes the system more versatile for functionalization and bioconjugation with different bioreceptors, such as antibodies or enzymes, allowing for a wide range of applications. The gold shell also enhances the stability of the MNPs in biological fluids and reduces the potential for aggregation. Compared to other electrochemical immunoassays reported in the literature, the developed magnetic immunoassay is cost-effective, portable, and user-friendly, without requiring expensive equipment. Additionally, the assay developed in this work demonstrated an acceptable LOD for *E. coli* O157:H7 within a short assay time, which is comparable with the works reported earlier. The benefits of this magnetic immunoassay include the elimination of complex immobilization procedures, ease of use, and analysis in a cost-effective assay, making it practical for the detection of *E. coli* O157:H7. Furthermore, this approach can be adapted for the detection of other pathogens by utilizing different bacterium-specific bio-elements.

## 4. Conclusions

In this work, an electrochemical magnetic immunoassay for the rapid analysis of *E. coli* O157:H7 bacteria was developed by using core–shell Fe_3_O_4_@Au MNPs. The core–shell MNPs were successfully synthesized by coating magnetic Fe_3_O_4_ cores with a gold shell through the reduction of Au(III) into Au(0) on the surface of the cores promoted by oleylamine. The resulting Fe_3_O_4_@Au MNPs were functionalized with a mixture of 6-mercapto-1-hexanol and thioctic acid in ethanol. These magnetic platforms were used in a sandwich-type assay and were easily held on the surface of an SPCE with an external magnet.

The specific capture antibody was covalently bioconjugated to the carboxylated self-assembled monolayer on the surface of Fe_3_O_4_@Au MNPs, while a detection antibody and a secondary HRP-labeled antibody were also used in the assay. Chronoamperometric measurements allowed efficient bacteria analysis in the linear range between 20 and 2 × 10^6^ CFU/mL. Recovery analysis demonstrated that at least 2 × 10^2^ CFU/mL of *E. coli* could be efficiently detected in a milk sample. The developed immunoassay offers a cost-effective, portable, and user-friendly solution, without the need for expensive equipment. It allows the detection of *E. coli* O157:H7 in a short time (1 h) with an acceptable LOD, which is a better performance when compared to other reported electrochemical assays. The immunoassay eliminates complex immobilization procedures, making it practical for the food industry to perform fast and reliable analysis. Moreover, it can be adapted for the detection of other pathogens by utilizing different bacterium-specific bio-elements.

## Figures and Tables

**Table 1:** Average particle size of the Fe_3_O_4_ and Fe_3_O_4_@Au MNPs determined by TEM and DLS.

	Average Particle Size			
MNP	*D*_TEM_ (nm)	*σ*_(TEM)_ ^c^	*D*_DLS_ (nm) ^d^	PdI ^e^
Fe_3_O_4_	5.4 ^a^	1.0	5.6	0.145
Fe_3_O_4_@Au	13.7 ^b^	1.9 nm	15.7	0.076

^a^ Estimated by TEM assuming a log-normal particle size distribution; ^b^ Estimated by TEM assuming a Gaussian particle size distribution; ^c^ Standard deviation; ^d^ Solvodynamic particle size estimated by DLS (dispersion in toluene); and ^e^ Polydispersity index value.

**Table 2:** Results of the recovery studies obtained in the analysis of *E. coli* O157:H7 in spiked milk samples and respective coefficients of variation.

[*E. coli*] Added (CFU/mL)	[*E. coli*] Found (CFU/mL)	Recovery (%) ^a^	CV (%) ^b^
0	<LOD		
2 × 10^1^	<LOD		
2 × 10^2^	2.5 × 10^2^	125	2.2
2 × 10^3^	2.3 × 10^3^	115	1.7

^a^ Recovery (%) = detected concentration/spiked concentration × 100; ^b^ coefficient of variation.

**Table 3:** Comparison of the main characteristics of the developed magnetic immunoassay with other electrochemical methods for the detection of *E. coli*.

Target Bacteria	Transducer	Modification/Platform	Label/Redox Probe	Technique	Assay Time	LOD (CFU/mL)	Ref.
*E. coli*	Glassy carbon electrode	-	[Fe(CN)_6_]^4−^	Chronoamperometry	<5 min	1 × 10^4^	[15]
*E. coli* O157:H7	SPCE	Magnetic nanobeads	Label-free	EIS ^a^	<1 h	1 × 10^4^	[27]
*E. coli*	Gold disk electrode	-	Label-free	EIS ^a^	<20 min	6 × 10^3^	[28]
*E. coli* O157:H7	Graphene paper electrode	AuNPs	Label-free	EIS ^a^	-	1.5 × 10^2^	[29]
*E. coli* O157:H7	Screen-printed interdigitated microelectrodes	-	[Fe(CN)_6_]^3−/4−^	EIS ^a^	<1 h	1 × 10^2^	[30]
*E. coli* O157:H7	SAM-modified gold electrodes	AuNPs	[Fe(CN)_6_]^3−/4−^	EIS ^a^	-	1 × 10^2^	[31]
*E. coli* O157:H7	Nanoporous membrane	Magnetic beads	Label-free	EIS ^a^	-	10	[32]
*E. coli* O157:H7	ITO ^c^	Plg-IONPs ^b^	[Fe(CN)_6_]^3−/4−^	EIS ^a^	-	3	[33]
*E. coli* O157:H7	SPCE	Fe_3_O_4_@Au MNPs	HRP	Chronoamperometry	1 h	20	This work

^a^ Electrochemical impedance spectroscopy; ^b^ poly(lactic-co-glycolic acid) coated iron oxide nanoparticles; and ^c^ indium tin oxide.

## Data Availability

Not applicable.
